# Interrelation Between Dilatation Rate and Displacement Speed in Premixed Turbulent Flames

**DOI:** 10.1007/s10494-025-00722-y

**Published:** 2025-12-15

**Authors:** Vishnu Mohan, Ruslan Khamedov, Hong G. Im, Markus Klein, Nilanjan Chakraborty

**Affiliations:** 1 School of Engineering, Newcastle University, Claremont Road, Newcastle-Upon-Tyne, NE1 7RU UK; 2 Clean Combustion Research Centre, King Abdullah Institute of Science and Technology, Thuwal, Kingdom of Saudi Arabia; 3 Department of Aerospace Engineering, University of the Bundeswehr Munich, Neubiberg, Germany

**Keywords:** Dilatation rate, Density-weighted displacement speed, Reaction rate, Turbulent premixed flames, Direct Numerical Simulation

## Abstract

The interrelation between displacement speed and dilatation rate in premixed turbulent flames has been analysed using three-dimensional Direct Numerical Simulation (DNS) data. The study focuses on statistically planar turbulent premixed flames with single-step chemistry at a unity Lewis number, representing stoichiometric methane-air premixed combustion, and detailed chemistry NH_3_-air premixed flames with an equivalence ratio of 1.2. It has been shown analytically that the dilatation rate based on fluid velocity is proportional to the product of the density-weighted displacement speed and the reaction progress variable gradient for unity Lewis number adiabatic conditions. This is demonstrated using unity Lewis number single-step chemistry DNS data and detailed chemistry DNS results of NH_3_-air premixed flames with an effective Lewis number close to unity. The study reveals that density-weighted displacement speed positively correlates with dilatation rate based on fluid velocity, though the relationship is non-linear due to the non-linear relationship between density-weighted displacement speed and the reactive scalar gradient magnitude. The density-weighted displacement speed and the dilatation rate based on flame propagation velocity are negatively correlated for the flames belonging to the wrinkled/corrugated flamelets regime, while their joint PDFs in thin reaction zones regime flames show both positive and negative correlation branches. The curvature stretch rate primarily causes these positive and negative correlation branches, a tendency that strengthens with an increase in Karlovitz number. Additionally, the mean dilatation rate in premixed turbulent flames is found to be proportional to the mean reaction rate in Reynolds Averaged Navier-Stokes simulations, suggesting that the mean dilatation rate could be linked to mean reaction rate closure.

## Introduction

The thermal expansion as a result of heat release rate in premixed turbulent flames is often quantified in terms of dilatation rate. Self-propagation is an intrinsic characteristic of premixed flames. This is characterised by the speed at which the flame propagates normal to itself with respect to the background fluid motion, and this speed is referred to as the flame displacement speed $\:{S}_{d}$. The flame displacement speed is affected by the flame surface curvature and tangential strain rate for turbulent premixed flames, and it was analysed in detail in several previous studies using Direct Numerical Simulation (DNS) data (e.g. Gran et al. 1996; Peters et al. 1998; Echekki and Chen 1999; Chen and Im 1998, 2000; Im and Chen 2002; Chakraborty and Cant 2004, 2005, 2006; Chakraborty 2007; Klein et al., 2006; Chakraborty et al., 2007; Han and Huh 2008; Chakraborty et al. 2011, 2022; Nivarti and Cant 2019; Herbert et al. 2020; Dave and Chaudhuri 2020; Keil et al. 2021a, b; Ozel-Erol et al., 2021; Yuvraj et al., 2022 as examples of some key references). The curvature, strain rate and stretch rate (which includes both tangential strain rate and flame curvature effects) dependences of displacement speed have also been analysed for premixed flames based on analytical (Buckmaster 1976; Clavin and Williams, 1982; Williams 1985; Clavin and Graña-Otero, 2011; Giannakopoulos et al. 2015) and experimental (Renou et al. 1998; Hartung et al. 2009; Peterson et al. 2019; Skiba et al. 2024) means. An analytical study by Buckmaster (1976) conjectured that the interrelation between dilatation rate and displacement speed could provide some insights related to local flame quenching, which might not be readily obtained from the stretch rate dependences of displacement speed. Notwithstanding the possibility of flame quenching, the interrelation between dilatation rate and flame displacement speed in premixed turbulent flames has not been analysed in detail. This aspect is addressed in the present analysis by considering a three-dimensional DNS dataset of statistically planar turbulent premixed flames with a range of Karlovitz numbers. The discussion of the interrelation between dilatation rate and flame displacement speed is not addressed sufficiently in the existing literature and the present analysis addresses this gap.

The dilatation rate provides the fractional change of volume with respect to time, but the choice of the reference frame can alter the definition of the dilatation rate. For example, the dilatation rate evaluated based on the inertial frame is different to the dilatation rate evaluated using the reference frame attached to the flame. In the present analysis, dilatation rates in the aforementioned reference frames and the density-weighted flame displacement speed are extracted from DNS data to address the following main objectives of the present analysis:

- To demonstrate the correlation between the dilatation rate and density-weighted displacement speed across the flame front for a range of different Karlovitz numbers for two different frames of reference and different thermochemical treatments.
- To provide physical explanations for the observed behaviour in (a) and indicate the modelling implications.

The mathematical background and numerical implementation related to the DNS database used for the current analysis are provided in the next two sections. The following section will deal with the presentation of the results and their discussions. The main findings are summarised and conclusions are drawn in the final section of this paper.

## Mathematical Background

In premixed flames, the scalar field is often characterised in terms of a reaction progress variable $\:c$, which can be defined based on a suitable species mass fraction $\:{Y}_{\alpha\:}$ in the following manner: $\:c=({Y}_{\alpha\:}-{Y}_{\alpha\:,u})/({Y}_{\alpha\:,b}-{Y}_{\alpha\:,u})$ where $\:{Y}_{\alpha\:,u}$ and $\:{Y}_{\alpha\:,b}$ are the mass fractions of the species $\:\alpha\:$ in the fully unburned and fully burned gas, respectively. The reaction progress variable $\:c$ is defined in such a manner that it increases monotonically from 0 in the unburned reactants to 1.0 in fully burned products. The transport equation of $\:c$ takes the following form:

1 $$\:\rho\left(\partial{c}/\partial{t}+{u}_{j}\partial{c}/\partial{x}_{j}\right)=\dot{\omega}+\partial\left(\rho{D}\partial{c}/\partial{x}_{j}\right)/\partial{x}_{j}$$

where $\:\rho\:$ is the density, $\:{u}_{j}$ is the $\:{j}^{th}$component of velocity, $\:\dot{\omega\:}$ is the reaction rate of reaction progress variable and $\:D$ is the reaction progress variable diffusivity. Equation 1 can be recast for a given $\:c$ isosurface with respect to a reference frame attached to a particular isosurface in the following manner:

2 $$\:Dc/Dt=\partial\:c/\partial\:t+{V}_{j}\partial\:c/\partial\:{x}_{j}=0$$

Here, $\:{V}_{j}={u}_{j}+{S}_{d}{N}_{j}$ is the $\:{j}^{th}$ component of the flame propagation velocity with $\:{N}_{j}=-(\partial\:c/\partial\:{x}_{j})/|\nabla\:c|$ being the $\:{j}^{th}$ component of the flame normal vector. Using the expression of $\:{V}_{j}$, Eq. 2 can be reformulated as follows:

3 $$\:\partial\:c/\partial\:t+{u}_{j}\partial\:c/\partial\:{x}_{j}={S}_{d}|\nabla\:c|$$

A comparison between Eqs. 1 and 3 reveals that $\:{S}_{d}$ for a given $\:c$ isosurfaces is given as:

4 $$\:{S}_{d}=\left[\dot{\omega\:}+\partial\:\left(\rho\:D\partial\:c/\partial\:{x}_{j}\right)/\partial\:{x}_{j}\right]/\rho\:|\nabla\:c|$$

It can be seen from Eq. 4 that density change due to combustion can affect the magnitude of displacement speed depending on the choice of the reaction progress variable. Thus, it is often useful to consider the density-weighted displacement speed $\:{S}_{d}^{*}=\rho\:{S}_{d}/{\rho\:}_{0}$ where $\:{\rho\:}_{0}$ is the unburned gas density. Moreover, $\:{S}_{d}^{*}$ is fundamentally important from the perspective of turbulent premixed flame modelling (Boger et al. 1998; Han and Huh 2008; Chakraborty and Cant 2007) and therefore, $\:{S}_{d}^{*}$ instead of $\:{S}_{d}$ is used in the current paper for the foregoing analysis.

In order to analyse the interrelation of dilatation rate and displacement speed, it is worthwhile to express density $\:\rho\:$ in the following manner: $\:\rho\:={\rho\:}_{0}/(1+\tau\:\theta\:)$ under the assumption of the constant mean molecular weight (Bray 1980) where $\:\theta\:=(T-{T}_{0})/({T}_{ad}-{T}_{0})$ and $\:\tau\:=({T}_{ad}-{T}_{0})/{T}_{0}$ are the non-dimensional temperature and heat release parameter, respectively with $\:T,{T}_{0}$ and $\:{T}_{ad}$ being the dimensional temperature, unburned gas temperature and adiabatic flame temperature, respectively. For unity Lewis number globally adiabatic low Mach number premixed flames $\:\theta\:$ can be substituted by $\:c$ (Bray 1980). Using $\:\rho\:={\rho\:}_{0}/(1+\tau\:c)$ in the mass conservation equation (i.e., $\:\partial\:\rho\:/\partial\:t+\partial\:\left(\rho\:{u}_{j}\right)/\partial\:{x}_{j}=0$) yields:

5 $$\:\partial\:{u}_{j}/\partial\:{x}_{j}=\tau\:{S}_{d}^{\mathrm{*}}|\nabla\:c|$$

Moreover, the dilatation in the reference frame attached to the flame can be expressed for unity Lewis number flames (Dopazo et al. 2015, 2016) as:

6 $$\:\partial\:{V}_{j}/\partial\:{x}_{j}=\partial\:{u}_{j}/\partial\:{x}_{j}+\partial\:\left({S}_{d}{N}_{j}\right)/\partial\:{x}_{j}=\tau\:{S}_{d}^{\mathrm{*}}\left|\nabla\:c\right|+\partial\:\left[{S}_{d}^{*}\left(1+\tau\:c\right){N}_{j}\right]/\partial\:{x}_{j}$$

Equations 5 and 6 indicate that the dilatation rate is closely related to $\:{S}_{d}^{\mathrm{*}}$ and $\:|\nabla\:c|$. For steady-state unstretched laminar premixed flame both $\:{V}_{j}$ and $\:\partial\:{V}_{j}/\partial\:{x}_{j}$ vanish and $\:{S}_{d}^{*}={S}_{L}$ is obtained. Moreover, $\:\partial\:{u}_{j}/\partial\:{x}_{j}$ vanishes wherever $\:{S}_{d}^{\mathrm{*}}|\nabla\:c|$ assumes a zero value which is obtained either for unburned or for fully burned gases or in the case of flame quenching. Although Eqs. 5 and 6 are derived based on unity Lewis number assumption, it is expected that the qualitative behaviour of the interrelation between $\:{S}_{d}^{\mathrm{*}}$ and dilatation rate will remain unchanged in the context of multi-step chemistry when the effective Lewis number remains close to unity. Thus, the interrelation between dilatation rate and $\:{S}_{d}^{\mathrm{*}}$ in statistically planar turbulent premixed flames has been analysed based on (i) 3D DNS data for simple chemistry unity Lewis number characteristic of stoichiometric CH_4_-air flames and (ii) 3D DNS data for rich $\:\mathrm{N}{\mathrm{H}}_{3}-$air detailed chemistry flames which have effective Lewis number close to unity. The details of these DNS datasets are presented in the next section.

## Numerical Implementation

The analysis is based on two different datasets: first, an existing DNS database of statistically planar turbulent premixed flames using single-step Arrhenius type irreversible chemistry with unity Lewis number characteristic of stoichiometric methane-air combustion under atmospheric pressure and unburned gas temperature of $\:{T}_{0}=415K$ (which yields an unstretched laminar burning velocity $\:{S}_{L}$ of 0.68 m/s) is considered for this analysis (Kasten et al. 2022a, b; Klein and Chakraborty, 2024). This database was generated using a well-known code SENGA+ (Jenkins and Cant 1999). In SENGA+, high order finite-difference and high-order Runge-Kutta schemes are used for spatial discretisation and explicit time advancement, respectively. A standard pseudo-spectral methodology is used to initialise the turbulent velocity fluctuations for prescribed values of root-mean-square (rms) velocity to laminar burning and integral length scale to flame thickness ratios (i.e., $\:{u}^{{\prime\:}}/{S}_{L}$ and $\:l/{\delta\:}_{th}$ with $\:{\delta\:}_{th}=({T}_{ad}-{T}_{0})/{\mathrm{max}\left|\nabla\:T\right|}_{L}$ being the thermal flame thickness) following a prescribed Passot-Paquet spectrum (Passot and Paquet, 1987) using a well-known pseudo-spectral method (Rogallo 1981). The scalar field is initialised by a steady unstretched laminar premixed flame solution. The flame-turbulence interaction takes place under decaying turbulence for this database. The mean direction of flame propagation is taken to align with $\:x-$direction and boundaries in this direction are taken to be partially non-reflecting. These boundaries are specified following Navier-Stokes characteristic boundary conditions (NSCBC) (Poinsot and Lele 1992). The linear relaxation parameter for pressure for the partially non-reflecting boundary is taken to be $\:{K}_{r}=s\left(1-{M}^{2}\right)a/L$ with $\:s=0.25$ as recommended by Poinsot and Lele (1992), $\:M$ is the maximum Mach number of the flow, $\:a$ is the acoustic speed and $\:L$ is the domain length in the direction of the boundary. The transverse boundaries in $\:y-$ and $\:z-$directions are taken to be periodic, and no thermodynamic pressure drift within the domain was observed during the simulation. For these cases the specific heats, thermal conductivity and density-weighted diffusivity are taken to be constant, and thus all the assumptions behind Eq. 5 are met for this database. The domain size for these simulations is taken to be $\:45.75{\delta\:}_{th}\times\:45.75{\delta\:}_{th}\times\:45.75{\delta\:}_{th}$. The attributes of the simple chemistry DNS cases considered here are listed in Table 1 where the uniform Cartesian grid size to discretise the domain, heat release parameter $\:\tau\:$, Damköhler number $\:Da=l{S}_{L}/{u}^{{\prime\:}}{\delta\:}_{th}$ and Karlovitz number $\:Ka={\left({u}^{{\prime\:}}/{S}_{L}\right)}^{3/2}{\left(l/{\delta\:}_{th}\right)}^{-1/2}$ are listed along with the values of $\:{u}^{{\prime\:}}/{S}_{L}$ and $\:l/{\delta\:}_{th}$. Case A represents the wrinkled flamelets regime, whereas cases B and C represent the thin reaction zones regime combustion (Peters 2000) for the $\:Ka$ values reported in Table 1. For the sake of completeness, Karlovitz number based on the Zel’dovich flame thickness (i.e., $\:\delta\:={\alpha\:}_{T0}/{S}_{L}={\nu\:}_{0}/\left(Pr{S}_{L}\right)$ with $\:{\alpha\:}_{T0},{\nu\:}_{0}$ and $\:Pr$ being the thermal diffusivity, kinematic viscosity and Prandtl number in the unburned gas, respectively) $\:K{a}_{\delta\:}={\left({u}^{{\prime\:}}/{S}_{L}\right)}^{3/2}{\left(l/\delta\:\right)}^{-1/2}$ and Karlovitz number $\:K{a}_{\eta\:}={\left({\delta\:}_{th}/\eta\:\right)}^{2}$ based on the Kolmogorov length scale $\:\eta\:$ and turbulent Reynolds number $\:R{e}_{t}={u}^{{\prime\:}}l/{\nu\:}_{0}$ are listed in Table 2 for the cases considered here. The grid spacing ensures 11 grid points within the thermal flame thickness $\:{\delta\:}_{th}.\:$The simulations have been continued for $\:{t}_{sim}=\mathrm{m}\mathrm{a}\mathrm{x}({\delta\:}_{th}/{S}_{L},2l/u^{\prime\:})$ and by that time the volume-integrated values of kinetic energy and burning rate reached a quasi-stationary state. Interested readers are referred to Kasten et al. (2022a, b) and Chakraborty and Klein (2024) for further information regarding this database.

**Table 1:** Grid size and non-dimensional parameters for all cases studied

Case	Chemical mechanism	$\:{\boldsymbol{u}}^{\boldsymbol{{\prime\:}}}/{\boldsymbol{S}}_{\boldsymbol{L}}$	$\:\boldsymbol{l}/{\boldsymbol{\delta\:}}_{\boldsymbol{t}\boldsymbol{h}}$	$\:{\boldsymbol{N}}_{\boldsymbol{x}}\times\:{\boldsymbol{N}}_{\boldsymbol{y}}\times\:{\boldsymbol{N}}_{\boldsymbol{z}}$	$\:\boldsymbol{\tau\:}$	$\:\boldsymbol{K}\boldsymbol{a}$	$\:\boldsymbol{D}\boldsymbol{a}$
A	Simple Chemistry	1.0	4.58	512×512×512	4.5	0.47	4.58
B	Simple Chemistry	5.0	4.58	512×512×512	4.5	5.2	0.92
C	Simple Chemistry	9.0	4.58	800$\:\times\:$400$\:\times\:$400	4.5	12.6	0.51
D	Detailed chemistry (NH_3_)	7.6	3.5	360$\:\times\:$180$\:\times\:$180	3.11	11.2	0.46
E	Detailed chemistry (NH_3_)	15.2	3.5	384$\:\times\:$208$\:\times\:$208	3.11	31.7	0.23

**Fig. 1:**
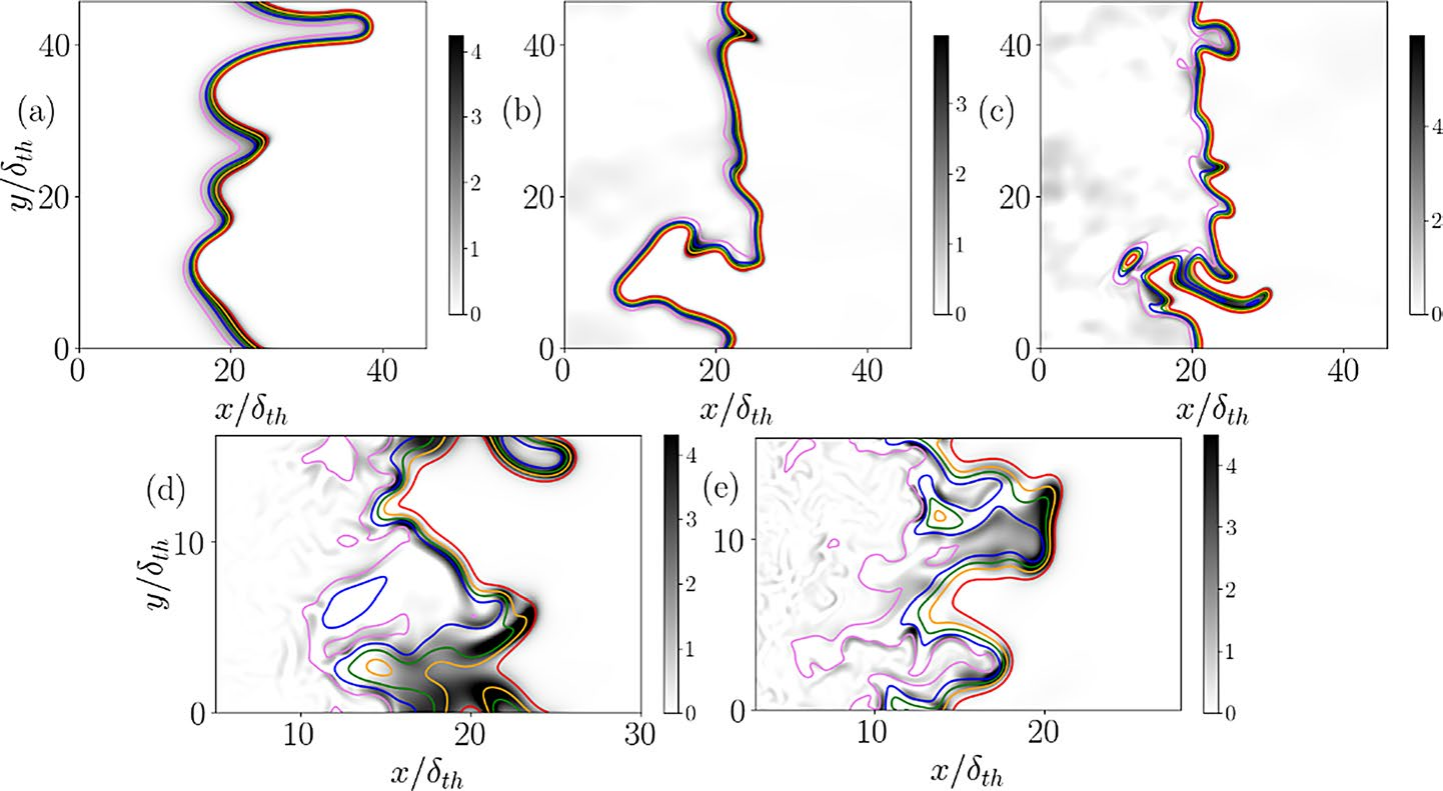
Contours of reaction progress variables $\:c$ (solid red line, $\:c=\mathrm{0.1,0.3,0.5,0.7,0.9}$ moving from left to right) and distribution of $\:\tau\:{S}_{d}^{\mathrm{*}}\left|\nabla\:c\right|\times\:{\delta\:}_{th}/{S}_{L}$ shown in grey color for cases A-E (**a**-**e**)

Secondly, a detailed chemistry DNS database of statistically planar NH_3_-air premixed flames preheated to 500 K with an equivalence ratio of 1.2 under atmospheric pressure (which yields an unstretched laminar burning velocity $\:{S}_{L}$ of 0.21 m/s) is generated using the KARFS code (KAUST Adaptive Reacting Flows Solver) (Hernandez-Perez et al., 2018). The KARFS code also uses a high-order finite difference scheme for spatial discretisation, whereas an operator-splitting technique is used for temporal advancement for tackling the numerical stiffness associated with the evaluation of chemical reaction rates. The chemical source term is evaluated using an implicit Runge-Kutta scheme, whereas an explicit high-order Runge-Kutta scheme is used for the time advancement of advection and diffusion terms. The boundaries in the $\:x-$direction are taken to be turbulent inflow and partially non-reflecting outflow, respectively and this direction is taken to align with the mean direction of flame propagation. The boundary conditions are specified using an improved version of the NSCBC technique (Yoo and Im 2007). The other boundaries are taken to be periodic and once again no pressure drift was observed in these simulations. These simulations also used $\:s=0.25$ and the transverse relaxation coefficient for Yoo and Im’s (2007) boundary condition is taken to be 0.05 following the recommendation of the original authors. The initialisation of turbulence and species fields followed the same procedures as that of the simple chemistry DNS database. A physical space forcing is employed in the unburned gas from the first 10% of the unburned gas side of the computational domain to maintain the turbulence level in the unburned gas, but the forcing is deactivated in the burned gas. A detailed chemical mechanism of NH_3_/H_2_/air combustion (Zhang et al. 2021) is simplified by the computational singular perturbation method to yield a skeletal mechanism involving 25 species and 175 reactions, which is used for the simulations conducted for this analysis. The domain size is taken to be $\:{32.636\delta\:}_{th}\times\:16.27{\delta\:}_{th}\times\:16.27{\delta\:}_{th}$ for case D and $\:{29.579\delta\:}_{th}\times\:15.98{\delta\:}_{th}\times\:15.98{\delta\:}_{th}$ for case E. The attributes of the detailed chemistry DNS cases are also listed in Table 1. The values of $\:K{a}_{\delta\:}$, $\:K{a}_{\eta\:}$ and $\:R{e}_{t}$ for these cases (i.e., cases D-E) are listed in Table 2. All the detailed chemistry cases belong to the thin reaction zones regime (Peters 2000). The grid spacing in the detailed chemistry DNS cases ensures at least 11 grid points within the thermal flame thickness $\:{\delta\:}_{th}$. The simulations have been continued until the desired values of $\:{u}^{{\prime\:}}/{S}_{L}$ and $\:l/{\delta\:}_{th}$ are obtained and both the turbulent burning velocity and flame surface area reached quasi-stationary state and this amounts to more than 20 eddy turnover times (i.e., $\:{t}_{sim}\ge\:20l/u{^\prime\:}$). Further detailed information on this database can be found elsewhere (Khamedov et al. 2024; Mohan et al. 2024).

**Table 2:** Alternative values of Karlovitz number and turbulent Reynolds number for all cases studied

Case	$\:\boldsymbol{K}{\boldsymbol{a}}_{\boldsymbol{\delta\:}}$	$\:\boldsymbol{K}{\boldsymbol{a}}_{\boldsymbol{\eta\:}}$	$\:\boldsymbol{R}{\boldsymbol{e}}_{\boldsymbol{t}}$
A	0.35	2.57	11.68
B	3.91	28.85	58.3
C	9.45	69.67	105.0
D	5.12	30.1	192.0
E	14.47	85.2	386.0

## Results and Discussion

**Fig. 2:**
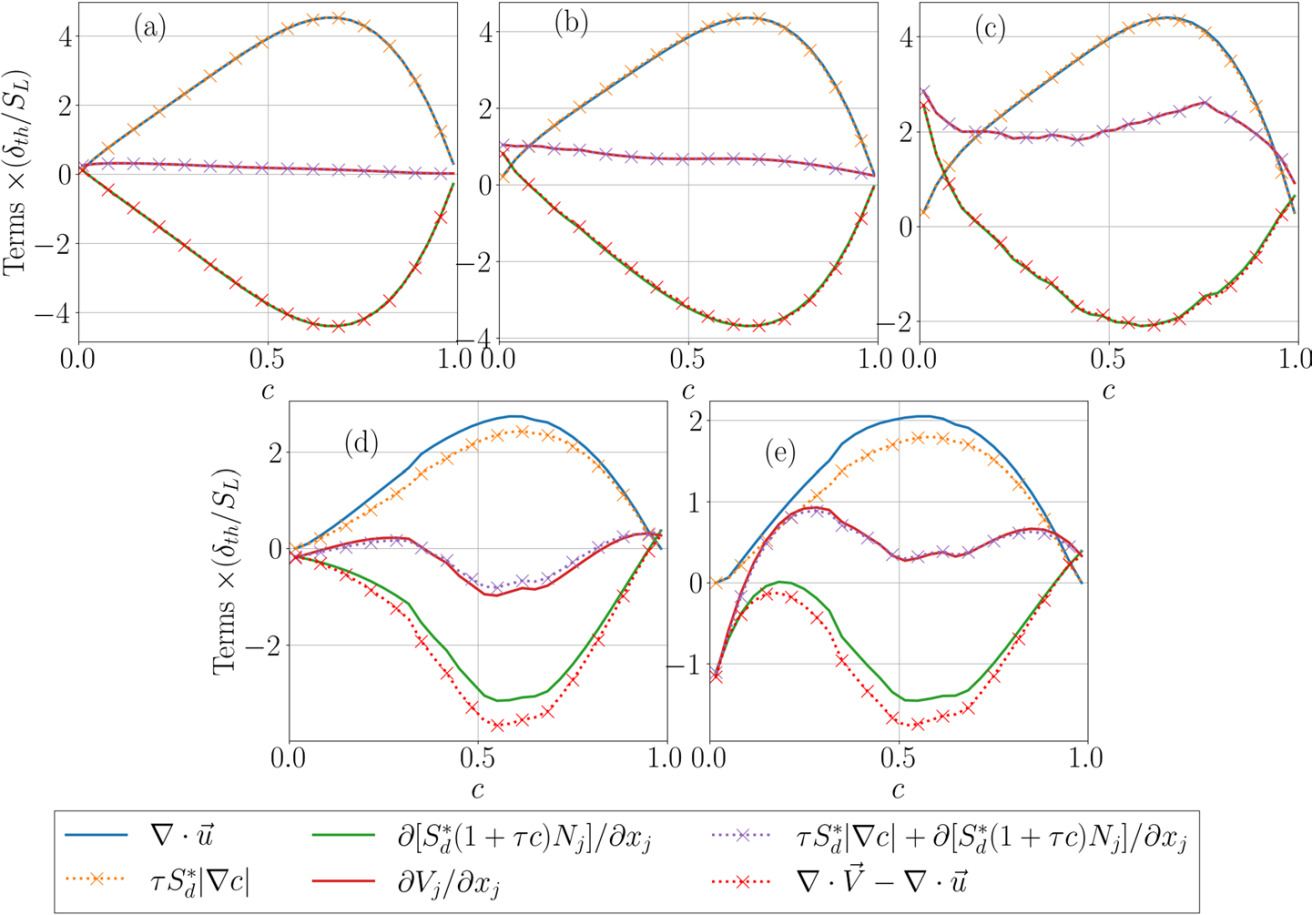
Distribution of the mean values of $$\:\{\partial\:{u}_{i}/\partial\:{x}_{i},\tau\:{S}_{d}^{\mathrm{*}}\left|\nabla\:c\right|,\partial\:\left[{S}_{d}^{*}\left(1+\tau\:c\right){N}_{j}\right]/\partial\:{x}_{j}, \partial\:{V}_{j}/\partial\:{x}_{j},$$$$\tau\:{S}_{d}^{\mathrm{*}}\left|\nabla\:c\right|+\partial\:\left[{S}_{d}^{*}\left(1+\tau\:c\right){N}_{j}\right]/\partial\:{x}_{j}$$$$,(\partial\:{V}_{i}/\partial\:{x}_{i}-\partial\:{u}_{i}/\partial\:{x}_{i})\}\times\:{\delta\:}_{th}/{S}_{L}$$ conditioned upon $$\:c$$ for cases A-E (**a**-**e**)

### Flame Morphology

The contours of reaction progress variable $\:c$ and distributions of $\:\tau\:{S}_{d}^{\mathrm{*}}\left|\nabla\:c\right|\times\:{\delta\:}_{th}/{S}_{L}$ in the central midplane for the simple chemistry cases (i.e., cases A-C) are shown in Figs. 1a-c, respectively, whereas the corresponding contours for reaction progress variable defined based on NH_3_ mass fraction (i.e., $\:c=({Y}_{N{H}_{3}}-{Y}_{N{H}_{3},u})/({Y}_{N{H}_{3},b}-{Y}_{N{H}_{3},u})$) and distributions of $\:\tau\:{S}_{d}^{\mathrm{*}}\left|\nabla\:c\right|\times\:{\delta\:}_{th}/{S}_{L}$ for the detailed chemistry cases (i.e., cases D and E) are shown in Fig. 1d and e, respectively. It can be seen from Fig. 1 that the contours of $\:c$ are parallel to each other for case A, whereas the contours of $\:c$ representing the preheat zone (i.e., $\:c<0.5$) are more wrinkled than the contours belonging to the reaction zone (i.e., $\:0.5<c<0.9$) in cases B-E and localised flame thickening is observed in these cases. This behaviour in cases B-E is the characteristic of the thin reaction zones regime combustion (Peters 2000) where the energetic, turbulent eddies penetrate into the preheat zone and cause unsteady fluctuations. However, the Kolmogorov length scale remains greater than the reaction zone thickness for cases B-E and thus the reaction zone remains unperturbed by the turbulence fluctuations in these cases. The qualitative nature of this conclusion remains unchanged if the reaction progress variable is defined based on a different species mass fraction (e.g., O_2_, H_2_O mass fractions) for the detailed chemistry DNS cases (i.e., cases D and E). In the subsequent analysis, the results for cases D and E are shown only for reaction progress variable definitions based on NH_3_ mass fraction. It can be seen from Figs. 1a-e that $\:\tau\:{S}_{d}^{\mathrm{*}}\left|\nabla\:c\right|$ assumes non-zero (mostly positive) values within the flame front but it vanishes both in unburned and burned gases.

### Variation of Mean Values for Relevant Quantities

The mean values of $$\:\{\partial\:{u}_{i}/\partial\:{x}_{i},\tau\:{S}_{d}^{\mathrm{*}}\left|\nabla\:c\right|,$$$$\partial\:{V}_{j}/\partial\:{x}_{j},\tau\:{S}_{d}^{\mathrm{*}}\left|\nabla\:c\right|$$$$+\partial\:\left[{S}_{d}^{*}\left(1+\tau\:c\right){N}_{j}\right]/\partial\:{x}_{j}\}\times\:{\delta\:}_{th}/{S}_{L}$$ conditioned upon $$\:c$$ are shown in Figs. 2a-e for cases A-E, respectively. It can be seen from Figs. 2a-e that the mean values of $$\:\partial\:{u}_{i}/\partial\:{x}_{i}$$ and $$\:\tau\:{S}_{d}^{\mathrm{*}}\left|\nabla\:c\right|$$ are identical for the unity Lewis number simple chemistry cases A-C, but these mean values remain close to each other but not identical for detailed chemistry NH_3_-air flames (i.e., cases D and E) for which the characteristic Lewis number (evaluated based on the formula suggested by Bechtold and Matalon 2001) is close to unity. Equation 5 was derived under the assumption of unity Lewis number adiabatic, low Mach number premixed combustion conditions where the mean molecular weight does not change significantly. All the cases considered here are low Mach number adiabatic cases. Cases A-C were designed in such a manner that all of the assumptions behind Eq. 5 are met in these cases, and detailed chemistry NH_3_-air flames (i.e., cases D and E) have a characteristic Lewis number (evaluated based on the formula suggested by Bechtold and Matalon 2001) close to unity. Moreover, for the stoichiometric CH_4_/air premixed flames (e.g., cases A-C) the mean molecular weights for the unburned and fully burned gases are equal to each other, whereas for the NH_3_-air flames with equivalence ratio of 1.2 (e.g., cases D and E) the mean molecular weight decreases marginally (~ 5%) in the burned gas and thus not all the assumptions behind the derivation of Eq. 5 are strictly met in cases D and E. Thus, the slight differences between the mean values of $$\:\partial\:{u}_{i}/\partial\:{x}_{i}$$ and $$\:\tau\:{S}_{d}^{\mathrm{*}}\left|\nabla\:c\right|$$ are expected for cases D and E.

Similarly, Figs. 2a-e show that the mean values of $\:\partial\:{V}_{j}/\partial\:{x}_{j}$ and $\:\tau\:{S}_{d}^{\mathrm{*}}\left|\nabla\:c\right|+\partial\:\left[{S}_{d}^{*}\left(1+\tau\:c\right){N}_{j}\right]/\partial\:{x}_{j}$ remain close to each other for all cases considered here. It is worth noting that the differences in the profiles of $\:\partial\:{u}_{i}/\partial\:{x}_{i},\tau\:{S}_{d}^{\mathrm{*}}\left|\nabla\:c\right|,\partial\:{V}_{j}/\partial\:{x}_{j},\tau\:{S}_{d}^{\mathrm{*}}\left|\nabla\:c\right|+\partial\:\left[{S}_{d}^{*}\left(1+\tau\:c\right){N}_{j}\right]/\partial\:{x}_{j}\}\times\:{\delta\:}_{th}/{S}_{L}$ between cases A-E arise because of differences in thermochemistry and turbulent flow conditions but this aspect is not the focus of the current analysis. The findings of Figs. 2a-e indicate Eqs. 5 and 6 serve as good approximations for premixed flames with unity Lewis number.

It is worth noting that the mean behaviours of $\:\partial\:\left[{S}_{d}^{*}\left(1+\tau\:c\right){N}_{j}\right]/\partial\:{x}_{j}$ and $\:\partial\:{V}_{j}/\partial\:{x}_{j}$ between simple and detailed chemistry cases originate due to the differences in spatial distributions of $\:{S}_{d}^{*}$ between these cases, which will not be discussed further in this paper as it is not relevant to the objective of the current analysis.

### Distributions of Dilatation Rates and Density-Weighted Displacement Speed

**Fig. 3:**
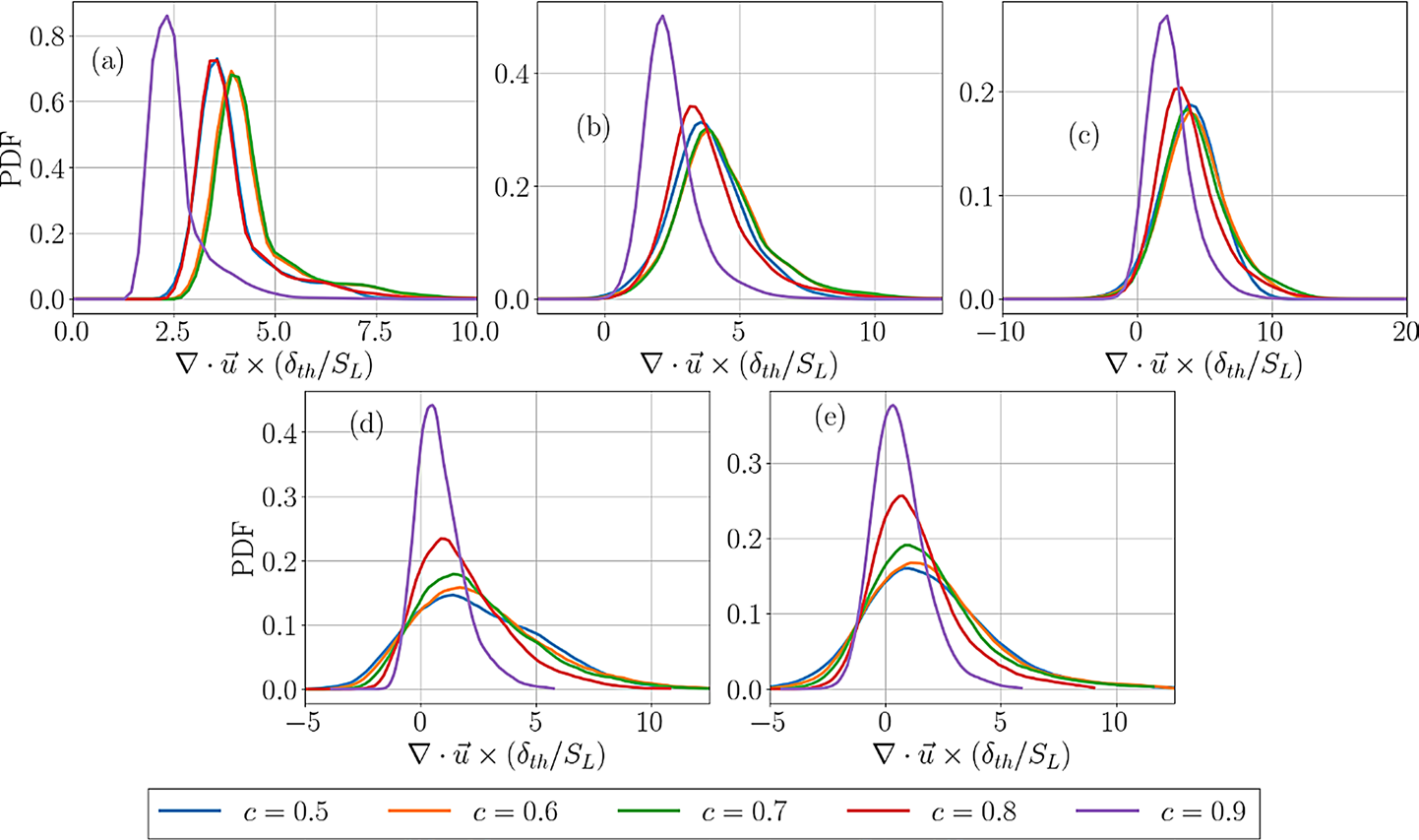
The PDFs $\:(\partial\:{u}_{i}/\partial\:{x}_{i})\times\:{\delta\:}_{th}/{S}_{L}$ for cases A-E for different $\:c$ values across the flame front

**Fig. 4:**
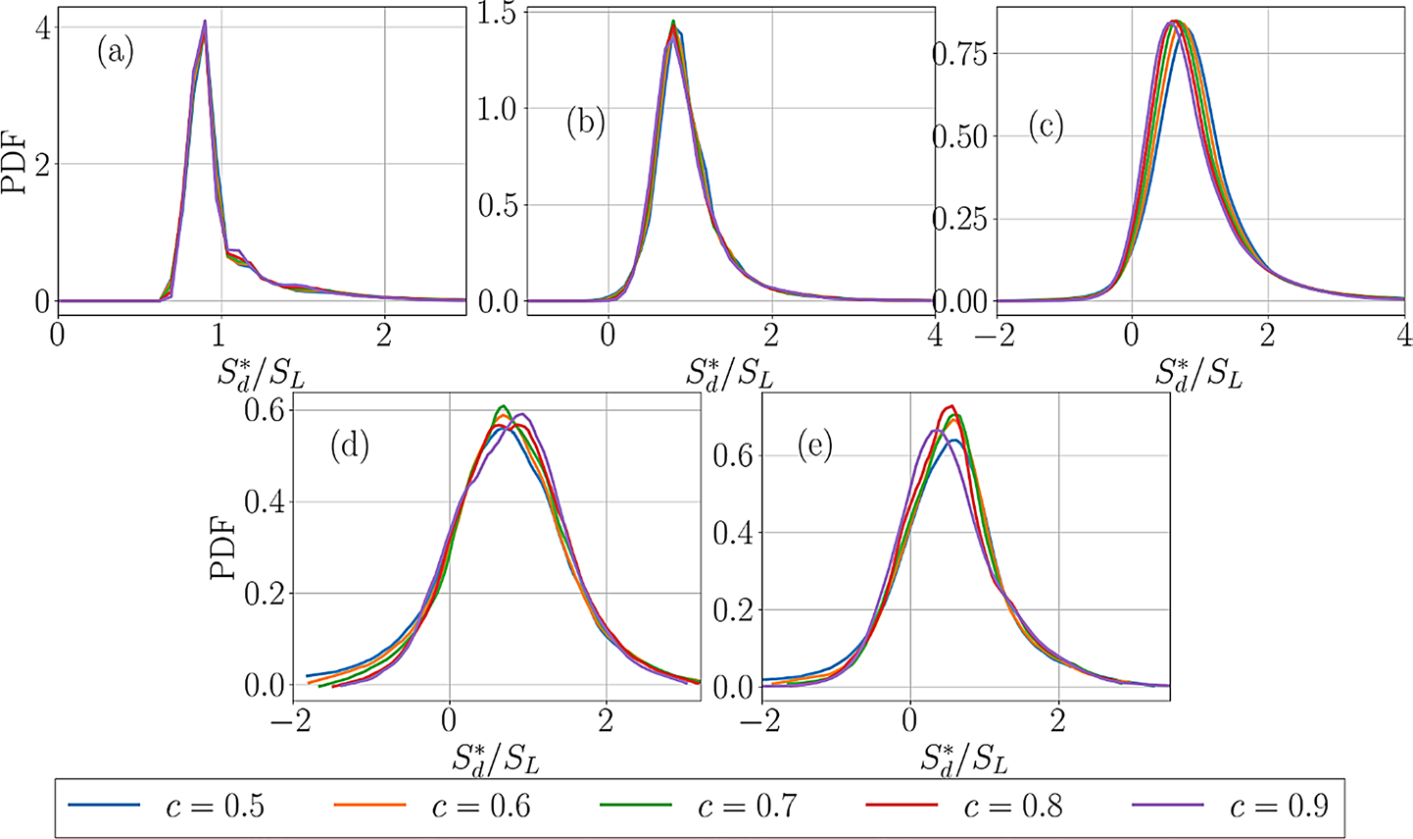
The PDFs of $\:{S}_{d}^{*}/{S}_{L}$ for cases A-E for different $\:c$ values across the flame front

The PDFs of normalised dilatation rate $\:(\partial\:{u}_{i}/\partial\:{x}_{i})\times\:{\delta\:}_{th}/{S}_{L}$ for cases A-E for different $\:c$ values across the flame front are shown in Figs. 3a-e. It can be seen from Fig. 3 that $\:\left(\partial\:{u}_{i}/\partial\:{x}_{i}\right)$ assumes predominantly positive values for all cases but there is a non-zero probability of obtaining negative values of $\:(\partial\:{u}_{i}/\partial\:{x}_{i})$ in cases B-E. To explain this behaviour, it is also worthwhile to consider the PDFs of normalised density-weighted displacement speed $\:{S}_{d}^{*}/{S}_{L}$ for the cases considered here, which are shown in Fig. 4. It can be seen from Fig. 4 that the flame in case A shows only positive values of $\:{S}_{d}^{*}$ but cases B-E exhibit finite probability of obtaining negative values. It has been discussed elsewhere (Gran et al. 1996; Chakraborty and Cant 2004, 2007) that a negative value of $\:{S}_{d}^{*}/{S}_{L}$ is obtained when the negative values of molecular diffusion rate $\:\nabla\:\cdot\:\left(\rho\:D\nabla\:c\right)$ overwhelm the positive reaction rate $\:\dot{\omega\:}$, and this tendency increases with an increase in Karlovitz number $\:Ka$ (Peters 2000). However, Fig. 4 shows that the most probable and the mean value of $\:{S}_{d}^{*}/{S}_{L}$ remain of the order of unity for all cases.

Equation 5 suggests that a negative $\:{S}_{d}^{*}$ value can lead to negative $\:\partial\:{u}_{i}/\partial\:{x}_{i}$. Therefore, a comparison between Figs. 3 and 4 reveals that the cases which show negative $\:{S}_{d}^{*}$ also exhibit negative $\:\partial\:{u}_{i}/\partial\:{x}_{i}$ values. Figures 3 and 4 also show the widths of the PDFs of $\:(\partial\:{u}_{i}/\partial\:{x}_{i})\times\:{\delta\:}_{th}/{S}_{L}$ and $\:{S}_{d}^{*}/{S}_{L}$ increase with an increase in turbulence intensity, which is a result of the increased range of strain rate, scalar gradient and curvature experienced by the flame with an increase in turbulence intensity, which, in turn, affect the values of $\:{S}_{d}^{*}$. The strain rate and curvature dependence of displacement speed were discussed elsewhere (Peters et al. 1998; Echekki and Chen 1999; Chen and Im 1998,2000; Im and Chen 2002; Chakraborty and Cant 2004, 2005, 2006; Chakraborty 2007; Klein et al., 2006; Chakraborty et al., 2007; Nivarti and Cant 2019; Herbert et al. 2020; Dave and Chaudhuri 2020; Chakraborty et al. 2022; Keil et al. 2021a, b; Ozel-Erol et al., 2021) in detail and thus are not presented here. The variation of $\:{S}_{d}^{*}$ in response to strain rate and curvature also leads to variations in $\:\partial\:{u}_{i}/\partial\:{x}_{i}$.

**Fig. 5:**
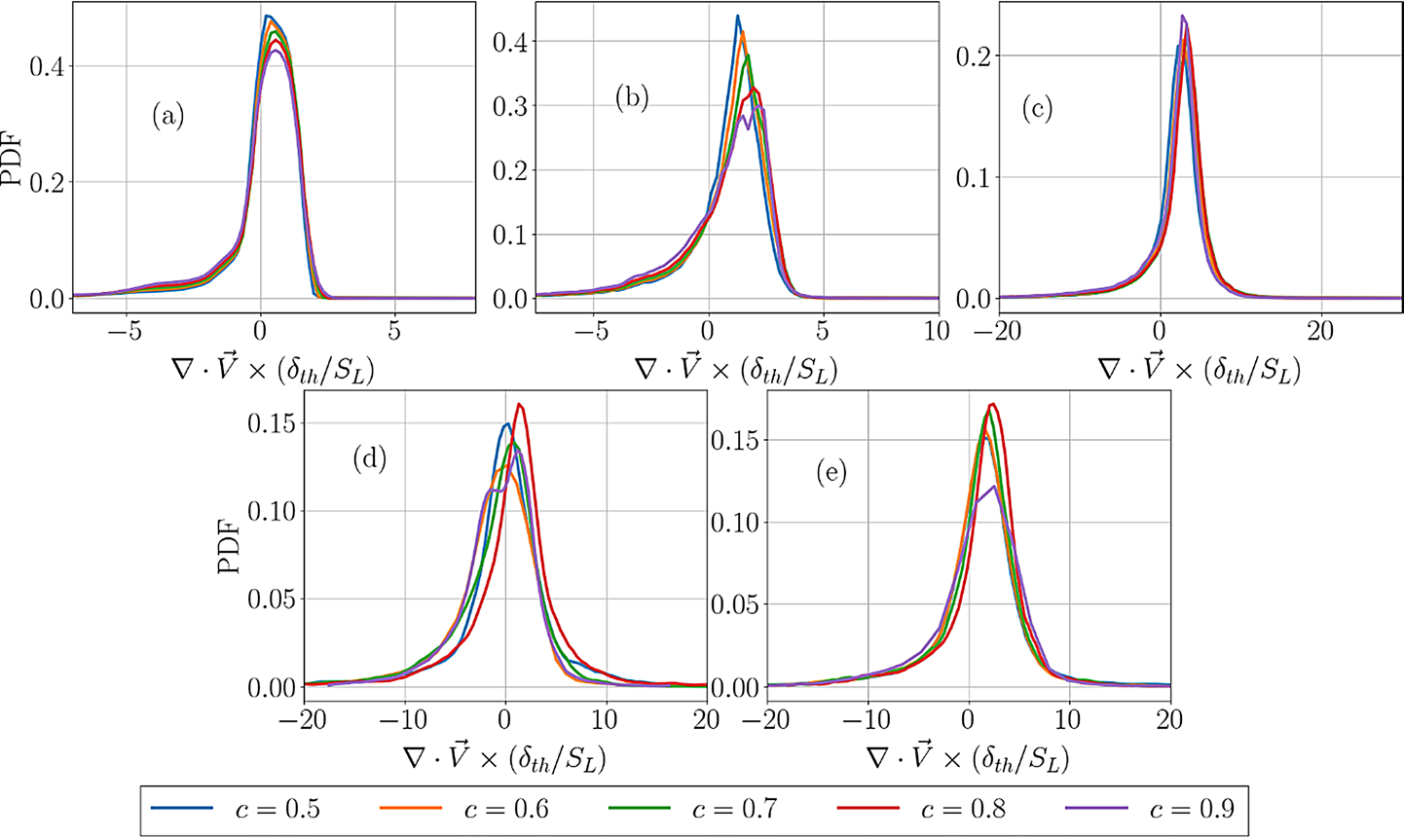
The PDFs of $\:(\partial\:{V}_{j}/\partial\:{x}_{j})\times\:{\delta\:}_{th}/{S}_{L}$ for cases A-E for different $\:c$ values

The PDFs of normalised dilatation rate $\:(\partial\:{V}_{j}/\partial\:{x}_{j})\times\:{\delta\:}_{th}/{S}_{L}$ in the reference frame attached to the flame for cases A-E for different $\:c$ values across the flame front are shown in Figs. 5a-e. A comparison between Figs. 3 and 5 reveals that the PDFs of $\:(\partial\:{V}_{j}/\partial\:{x}_{j})\times\:{\delta\:}_{th}/{S}_{L}$ show higher probability of negative values in comparison to the PDFs of $\:(\partial\:{u}_{i}/\partial\:{x}_{i})\times\:{\delta\:}_{th}/{S}_{L}$. The PDFs of $\:(\partial\:{V}_{j}/\partial\:{x}_{j})\times\:{\delta\:}_{th}/{S}_{L}$ exhibit long negative tails especially for the flames in the thin reaction zones regime. This can be explained by expressing the right-hand side of Eq. 6 as:

7 $$\:\frac{\partial\:{V}_{j}}{\partial\:{x}_{j}}=\frac{\partial\:{u}_{j}}{\partial\:{x}_{j}}+\frac{\partial\:\left({S}_{d}{N}_{j}\right)}{\partial\:{x}_{j}}=2\left({S}_{r}+{S}_{n}\right){\kappa\:}_{m}-4D{\kappa\:}_{m}^{2}+{N}_{j}(1+\tau\:c)\frac{\partial\:{S}_{d}^{\mathrm{*}}}{\partial\:{x}_{j}}$$

Here, $\:{\kappa\:}_{m}=0.5\nabla\:\cdot\:\overrightarrow{N}$ is the local flame curvature such that it assumes positive (negative) values when the flame surface is convex (concave) towards the reactants, and $\:{S}_{r}=\dot{\omega\:}/\left(\rho\:\right|\nabla\:c\left|\right)$ and $\:{S}_{n}=\overrightarrow{N}\cdot\:\nabla\:(\rho\:D\overrightarrow{N}\cdot\:\nabla\:c)/\left(\rho\:\right|\nabla\:c\left|\right)$ are the reaction and normal diffusion components of displacement speed (Peters et al. 1998; Echekki and Chen 1999). The term $\:2\left({S}_{r}+{S}_{n}\right){\kappa\:}_{m}$ assumes both positive and negative, whereas $\:-4D{\kappa\:}_{m}^{2}$ assumes deterministically negative values. The magnitude of the contribution of $\:{N}_{j}(1+\tau\:c)\partial\:{S}_{d}^{*}/\partial\:{x}_{j}$ remains small in comparison to $\:-4D{\kappa\:}_{m}^{2}$ especially for the thin reaction zones regime flames. Therefore, $\:(\partial\:{V}_{j}/\partial\:{x}_{j})\times\:{\delta\:}_{th}/{S}_{L}$ shows a long negative tail due to the dominant contribution of $\:(-4D{\kappa\:}_{m}^{2})$. The PDFs of $\:(\partial\:{V}_{j}/\partial\:{x}_{j})\times\:{\delta\:}_{th}/{S}_{L}$ widen from the unburned to the burned gas side due to an increase in the range of values obtained for $\:(-4D{\kappa\:}_{m}^{2})$ as a result of the increased magnitude of diffusivity with increasing $\:c$.

### Local Interdependence Between Dilatation Rates and Density-Weighted Displacement Speed

**Fig. 6:**
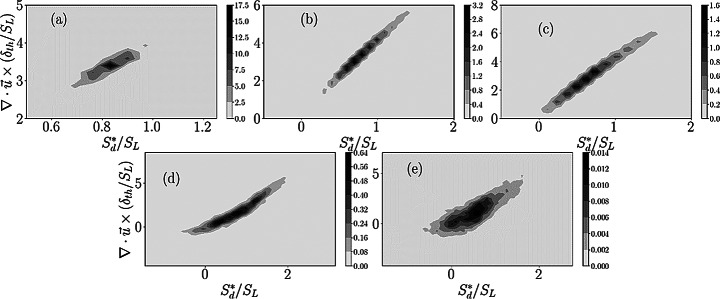
The contours of the joint PDFs between $\:{S}_{d}^{*}/{S}_{L}$ and $\:(\partial\:{u}_{j}/\partial\:{x}_{j})\times\:{\delta\:}_{th}/{S}_{L}$ for the $\:c=0.8$ isosurface. The correlation coefficients for (**a**-**e**) cases A-E are: 0.99,0.98,0.98, 0.94 and 0.85 respectively

At this point, the local interdependence between $\:{S}_{d}^{*}$ and dilatation rate is worth investigating. The contours of the joint PDFs between $\:{S}_{d}^{*}/{S}_{L}$ and $\:(\partial\:{u}_{j}/\partial\:{x}_{j})\times\:{\delta\:}_{th}/{S}_{L}$ for the $\:c=0.8$ isosurface are shown in Figs. 6a-e for cases A-E. The choice of $\:c=0.8$ is motivated by the fact that the maximum heat release rate for both unstretched laminar and turbulent flames for both the present simple and detailed chemistry DNS cases (see Chakraborty 2007 for simple chemistry DNS for the present thermochemistry and Shahsavari et al. 2025 for NH_3_/air premixed flames with $\:\phi\:=1.2$) occurs close to this value of $\:c$ and thus the $\:c=0.8$ isosurface can be considered as the flame surface in this analysis. However, the joint PDFs shown in Fig. 6 and the following figures are qualitatively similar for other isosurfaces of $\:c$ where the heat release rate effects are strong, which is not explicitly shown here for brevity.

**Fig. 7:**
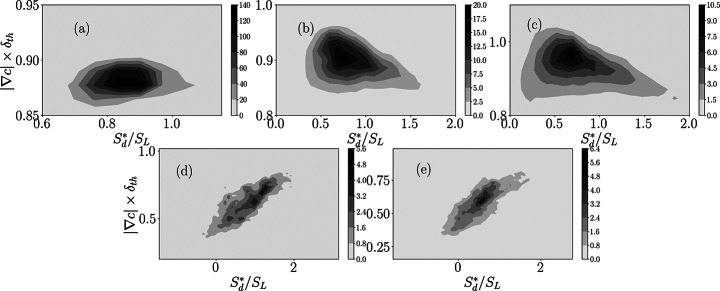
The contours of the joint PDFs between $\:{S}_{d}^{*}/{S}_{L}$ and $\:|\nabla\:c|\times\:{\delta\:}_{th}$ for the $\:c=0.8$ isosurface. The correlation coefficients for (**a**-**e**) cases A-E are: -0.80, -0.54, -0.45, 0.76 and 0.74 respectively

Equation 5 indicates that $\:(\partial\:{u}_{j}/\partial\:{x}_{j})$ is directly proportional to $\:{S}_{d}^{\mathrm{*}}|\nabla\:c|$ when all the underlying assumptions are met. It can be seen from Fig. 6 that $\:{S}_{d}^{*}/{S}_{L}$ and $\:(\partial\:{u}_{j}/\partial\:{x}_{j})\times\:{\delta\:}_{th}/{S}_{L}$are found to be positively correlated for all cases. This is in accordance with the expectation from Eq. 5 because $\:|\nabla\:c|$ is a positive semi-definite quantity (i.e., $\:|\nabla\:c|\ge\:0$). However, a careful examination of Fig. 6 reveals that the interdependence between $\:{S}_{d}^{*}/{S}_{L}$ and $\:(\partial\:{u}_{j}/\partial\:{x}_{j})\times\:{\delta\:}_{th}/{S}_{L}$ is non-linear in nature, which becomes more prominent for the cases with high turbulence intensities (i.e., $\:{u}^{{\prime\:}}/{S}_{L}\gg\:1$). Equation 5 should indicate that even when this relation is fully valid, the correlation between $\:{S}_{d}^{*}/{S}_{L}$ and $\:(\partial\:{u}_{j}/\partial\:{x}_{j})\times\:{\delta\:}_{th}/{S}_{L}$ depends on the interrelation between $\:{S}_{d}^{*}$ and $\:|\nabla\:c|$. Thus, this non-linear dependence between between $\:{S}_{d}^{*}/{S}_{L}$ and $\:(\partial\:{u}_{j}/\partial\:{x}_{j})\times\:{\delta\:}_{th}/{S}_{L}$ arises from the non-linear dependence between $\:|\nabla\:c|$ and $\:{S}_{d}^{*}$, which can be substantiated from the joint PDFs between $\:{S}_{d}^{*}/{S}_{L}$ and $\:|\nabla\:c|\times\:{\delta\:}_{th}$ for the $\:c=0.8$ isosurface presented in Fig. 7. This non-linear dependence between $\:|\nabla\:c|$ and $\:{S}_{d}^{*}$ arises principally due to the complex non-linear curvature dependence of $\:|\nabla\:c|$. This in turn induces non-linear $\:|\nabla\:c|$ dependence of the tangential diffusion component of density-weighted displacement speed $\:{S}_{t}^{*}=-2\rho\:D{\kappa\:}_{m}/{\rho\:}_{0}$. Moreover, an increase in $\:|\nabla\:c|$ acts to influence $\:{S}_{r}^{*}$ and $\:{S}_{n}^{*}$, which also contributes to the non-linear dependence between $\:|\nabla\:c|$ and $\:{S}_{d}^{*}$. The relative strengths of these mechanisms determine the net correlation between $\:{S}_{d}^{*}/{S}_{L}$ and $\:|\nabla\:c|\times\:{\delta\:}_{th}$, which are different between cases A-E, but these differences are not the subject of this analysis. However, it is worth mentioning that these differences arise due to the differences in strain rate and curvature dependences of $\:|\nabla\:c|$ between simple and detailed chemistry cases, which was demonstrated earlier by Keil et al. (2021a) for stoichiometric methane-air flames. It has also been discussed earlier that all the assumptions behind the derivation of Eq. 5 are identically met for cases A-C, whereas they are not strictly valid in cases D and E. This also contributes to the increased non-linear correlation between $\:{S}_{d}^{*}/{S}_{L}$ and $\:(\partial\:{u}_{j}/\partial\:{x}_{j})\times\:{\delta\:}_{th}/{S}_{L}$ in cases D-E in comparison to cases A-C. These effects could also affect the differences in correlations between $\:{S}_{d}^{*}/{S}_{L}$ and $\:(\partial\:{V}_{j}/\partial\:{x}_{j})\times\:{\delta\:}_{th}/{S}_{L}$ between simple chemistry and detailed chemistry cases considered here.

**Fig. 8:**
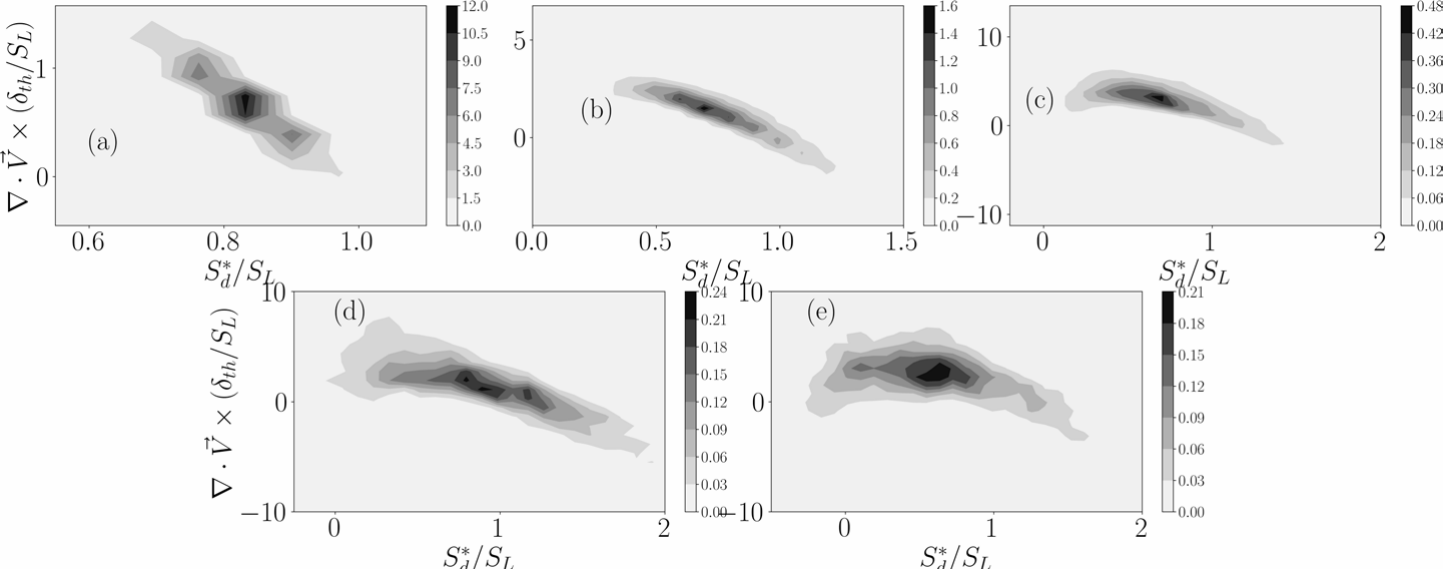
The contours of the joint PDFs between $\:{S}_{d}^{*}/{S}_{L}$ and $\:(\partial\:{V}_{j}/\partial\:{x}_{j})\times\:{\delta\:}_{th}/{S}_{L}$ for the $\:c=0.8$ isosurface. The correlation coefficients for (**a**-**e**) cases A-E are: -0.94, -0.86, -0.65, -0.44 and − 0.37 respectively

The joint PDF contours between $\:{S}_{d}^{*}/{S}_{L}$ and $\:(\partial\:{V}_{j}/\partial\:{x}_{j})\times\:{\delta\:}_{th}/{S}_{L}$ (i.e. in the reference frame attached to the flame) for the $\:c=0.8$ isosurface are shown in Figs. 8a-e for cases A-E. The quantities $\:{S}_{d}^{*}/{S}_{L}$ and $\:(\partial\:{V}_{j}/\partial\:{x}_{j})\times\:{\delta\:}_{th}/{S}_{L}$ are found to be negatively correlated for case A but both positive and negatively correlating branches can be seen for other cases and this tendency becomes increasingly prominent with an increase in Karlovitz number $\:Ka$. It has already been discussed that$\:\:-4D{\kappa\:}_{m}^{2}$ is the major contributor to $\:\partial\:{V}_{j}/\partial\:{x}_{j}$. This term is responsible for inducing both positive and negative correlating branches in the joint PDFs of $\:{S}_{d}^{*}/{S}_{L}$ and $\:(\partial\:{V}_{j}/\partial\:{x}_{j})\times\:{\delta\:}_{th}/{S}_{L}$ because $\:{S}_{d}^{*}/{S}_{L}$ and $\:{\kappa\:}_{m}$ are negatively correlated for these flames. The contribution of $\:-4D{\kappa\:}_{m}^{2}$ strengthens with increasing $\:Ka$ (Chen and Im 2000; Chakraborty 2007; Herbert et al. 2020; Chakraborty et al. 2022) and thus the appearance of both positive and negative correlation branches are prominent for the thin reaction zones regime flames. In the wrinkled/corrugated flamelets regime, $\:2\left({S}_{r}+{S}_{n}\right){\kappa\:}_{m}$ is the leading order contributor to $\:\partial\:{V}_{j}/\partial\:{x}_{j}$, which leads to a predominant negative correlation between $\:{S}_{d}^{*}/{S}_{L}$ and $\:(\partial\:{V}_{j}/\partial\:{x}_{j})\times\:{\delta\:}_{th}/{S}_{L}$ because $\:2\left({S}_{r}+{S}_{n}\right){\kappa\:}_{m}$ and $\:{S}_{d}^{*}$ remain positively and negatively correlated with $\:{\kappa\:}_{m}$, respectively.

### Modelling Implications

It is important to note that Eq. 5 is an exact expression under the assumption of unity Lewis number adiabatic, low Mach number premixed combustion conditions where the mean molecular weight does not change significantly. This suggests that the density-weighted displacement speed and dilatation rate are closely related in premixed turbulent flames. In fact, Eq. 5 can be averaged or filtered to obtain (Boger et al. 1998):

8 $$\:\overline{\left[\dot{\omega\:}+\nabla\:\cdot\:\left(\rho\:D\nabla\:c\right)\right]}=\overline{{\left(\rho\:{S}_{d}\right)}}_{s}{{\Sigma\:}}_{gen}={{\rho\:}_{0}\overline{\left({S}_{d}^{\mathrm{*}}\right)}}_{s}{{\Sigma\:}}_{gen}=({\rho\:}_{0}/\tau\:)\partial\:{\overline{u}}_{i}/\partial\:{x}_{i}$$

Here, the overbar suggests Reynolds averaging/LES filtering by a homogeneous filter (e.g. space-independent Gaussian filter) as appropriate, and $\:{{\Sigma\:}}_{gen}=\overline{|\nabla\:c|}$ is the generalised Flame Surface Density (FSD) and $\:{\overline{\left({Q}\right)}}_{s}=\overline{Q|\nabla\:c|}/\overline{|\nabla\:c|}$ is the surface-weighted value of a general quantity $\:Q$ (Boger et al. 1998). As the contribution of $\:\overline{\nabla\:\cdot\:(\rho\:D\nabla\:c)}$ is negligible in comparison to $\:\overline{\dot{\omega\:}}$ in RANS, Eq. 8 relates $\:\partial\:{\overline{u}}_{i}/\partial\:{x}_{i}$ with mean reaction rate $\:\overline{\dot{\omega\:}}$. Equation 8 can also be taken to be the exact expression subject to the above assumptions. The reasonable validity of Eq. 5 for the cases considered here has been demonstrated in Fig. 2. Thus, it is important to recognise that Eqs. 5 and 8 are mathematical identities for the aforementioned assumptions. For low Mach number conditions (e.g., $\:Ma\ll\:0.3,$ where $\:Ma$ is the Mach number) dilatation rate is expected to be negligibly small without chemical reactions. Equation 8 shows that this relation can be utilised to relate $\:\overline{\dot{\omega\:}}$ and $\:\partial\:{\overline{u}}_{i}/\partial\:{x}_{i}$, and it can potentially be utilised in low Mach number formulations under the assumption of unity Lewis number adiabatic conditions where the mean molecular weight does not change significantly. Moreover, Eq. 8 could be a useful diagnostic tool because it allows for determining the combined contribution of mean/filtered reaction rate and molecular diffusion rate based on dilatation rate.

The Reynolds averaged velocity components $\:{\overline{u}}_{i}$ can be expressed as (Bray et al. 1985):

9 $$\:{\overline{u}}_{i}={\overline{\left({u}_{i}\right)}}_{R}\left(1-\overline{c}\right)+\overline{c}{\overline{\left({u}_{i}\right)}}_{P}+O(1/Da)$$

where $\:{\overline{\left({u}_{i}\right)}}_{R}$ and $\:{\overline{\left({u}_{i}\right)}}_{P}$ are the *i*^*th*^ component of velocity conditional in reactants and products, respectively. The closures of $\:\overline{c}$, $\:{\overline{\left({u}_{i}\right)}}_{R}$ and $\:{\overline{\left({u}_{i}\right)}}_{P}$ have been discussed elsewhere (Chakraborty and Lipatnikov 2013) and thus are not repeated here. The current findings seem to suggest that Eqs. 8 and 9 can relate $\:\overline{\dot{\omega\:}}$ and $\:\partial\:{\overline{u}}_{i}/\partial\:{x}_{i}$ (or conditional mean velocities) for $\:Da\gg\:1$ flames (where $\:O(1/Da)$ is expected to be negligible) under unity Lewis number assumption for adiabatic low Mach number conditions. This suggests that the closures of $\:\overline{\dot{\omega\:}}$, $\:\overline{c}$, $\:{\overline{\left({u}_{i}\right)}}_{R}$ and $\:{\overline{\left({u}_{i}\right)}}_{P}$ are interlinked, and the closures of any three of these quantities could be utilised to obtain the remaining fourth quantity.

The results shown in the paper indicate that it is possible to obtain the following relation using : $$\:\overline{\rho\:}={\rho\:}_{0}/(1+\tau\:\stackrel{\sim}{\theta\:})$$ for the unity Lewis number flames (i.e., where $$\:\stackrel{\sim}{c}=\stackrel{\sim}{\theta\:}$$ can be assumed) in $$\:\partial\:\overline{\rho\:}/\partial\:t+\partial\:\left(\overline{\rho\:}{\stackrel{\sim}{u}}_{j}\right)/\partial\:{x}_{j}=0$$ using the transport equation of the Favre-averaged/filtered reaction progress variable $$\:\stackrel{\sim}{c}$$ (i.e., $$\:\partial\:\left(\overline{\rho\:}\stackrel{\sim}{c}\right)/\partial\:t+\partial\:\left(\overline{\rho\:}{\stackrel{\sim}{u}}_{j}\stackrel{\sim}{c}\right)/\partial\:{x}_{j}$$$$=\overline{\left[\dot{\omega\:}+\partial\:\left(\rho\:D\partial\:c/\partial\:{x}_{j}\right)/\partial\:{x}_{j}\right]}$$$$-\partial\:\left(\overline{\rho\:{u}_{j}^{{\prime\:}{\prime\:}}{c}^{{\prime\:}{\prime\:}}}\right)/\partial\:{x}_{j}$$):

10 $$\begin{aligned}\:\frac{{\rho\:}_{0}}{\tau\:}\frac{\partial\:{\stackrel{\sim}{u}}_{i}}{\partial\:{x}_{i}}&=\overline{\left[\dot{\omega\:}+\frac{\partial\:}{\partial\:{x}_{j}}\left(\rho\:D\frac{\partial\:c}{\partial\:{x}_{j}}\right)\right]}-\frac{\partial\:\left(\overline{\rho\:{u}_{j}^{{\prime\:}{\prime\:}}{c}^{{\prime\:}{\prime\:}}}\right)}{\partial\:{x}_{j}}\cr&={\overline{\left(\rho\:{S}_{d}\right)}}_{s}{{\Sigma\:}}_{gen}-\frac{\partial\:\left(\overline{\rho\:{u}_{j}^{{\prime\:}{\prime\:}}{c}^{{\prime\:}{\prime\:}}}\right)}{\partial\:{x}_{j}}\cr&={{\rho\:}_{0}\overline{\left({S}_{d}^{*}\right)}}_{s}{{\Sigma\:}}_{gen}-\frac{\partial\:\left(\overline{\rho\:{u}_{j}^{{\prime\:}{\prime\:}}{c}^{{\prime\:}{\prime\:}}}\right)}{\partial\:{x}_{j}}\end{aligned}$$

    

It is important to recognise that Eq. 10 is an exact equation as long as $\:\stackrel{\sim}{c}=\stackrel{\sim}{\theta\:}$ can be assumed under unity Lewis number condition irrespective of the value of Damköhler number. The right hand side of Eq. 10 are the two unclosed terms of the Favre-averaged/filtered transport equation, whereas the left hand side of Eq. 10 is closed in Reynolds Averaged Navier-Stokes (RANS)/Large Eddy Simulations (LES). Thus, Eq. 10 can be utilised to close the combined action of both the chemical source term and turbulent transport term in RANS/LES simulations subject to the unity Lewis number assumption (e.g., rich NH_3_-air premixed flames considered here) under low Mach number adiabatic conditions. Furthermore, a comparison between Eqs. 8 and 10 also provides the following relation for the turbulent transport term in the Favre-averaged/filtered reaction progress variable $\:\stackrel{\sim}{c}$ transport equation for the unity Lewis number condition:

11 $$\:\frac{\partial\:\left(\overline{\rho\:{u}_{j}^{{\prime\:}{\prime\:}}{c}^{{\prime\:}{\prime\:}}}\right)}{\partial\:{x}_{j}}=\frac{{\rho\:}_{0}}{\tau\:}\left[\frac{\partial\:{\overline{u}}_{i}}{\partial\:{x}_{i}}-\frac{\partial\:{\stackrel{\sim}{u}}_{i}}{\partial\:{x}_{i}}\right]$$

Equation 11 can also potentially have applications in the closure of turbulent/sub-grid transport terms due to $\:\overline{\rho\:{u}_{j}^{{\prime\:}{\prime\:}}{c}^{{\prime\:}{\prime\:}}}$.

## Conclusions

The interrelation between displacement speed and dilatation rate in premixed turbulent flames has been analysed in this paper based on three-dimensional Direct Numerical Simulation (DNS) data for statistically planar flames with single-step chemistry with unity Lewis number representing stoichiometric methane-air premixed flames and detailed chemistry NH_3_-air premixed flames with an equivalence ratio of 1.2. It has been demonstrated based on first principles that the dilatation rate based on fluid velocity is proportional to the product of the density-weighted displacement speed and the magnitude of the reaction progress variable gradient for unity Lewis number flames. The validity of the derived expression for unity Lewis number flames has been demonstrated for unity Lewis number single-step chemistry DNS data representing stoichiometric methane-air premixed flames and detailed chemistry DNS results of NH_3_-air premixed flames with effective Lewis number close to unity. It has been demonstrated that the density-weighted displacement speed is positively correlated with dilatation rate based on fluid velocity but the dependence between these quantities is non-linear, and this behaviour originates due to the non-linear relation between density-weighted displacement speed and the reactive scalar gradient magnitude. By contrast, the density-weighted displacement speed and the dilatation rate based on flame propagation velocity are found to be negatively correlated for the flame representing the wrinkled/corrugated flamelets but the joint PDFs of these quantities in the thin reaction zones regime flames show both positive and negative correlating branches. The contribution of the curvature stretch rate is principally responsible for the aforementioned positive and negative correlating branches in the joint PDF between the density-weighted displacement speed and the dilatation rate based on flame propagation velocity. This tendency is expected to strengthen with an increase in Karlovitz number. It has been demonstrated that the mean dilatation rate in premixed turbulent flames is proportional to the mean reaction rate in the context of Reynolds Averaged Navier-Stokes simulations which can potentially be utilised for modelling purposes. Furthermore, the dilatation rate based on Favre-averaged/filtered velocity can be linked to the combined effect of chemical source term and turbulent transport term in the Favre-averaged/filtered reaction progress variable transport equation under the assumption of unity Lewis number irrespective of Damköhler number value.

It is important to note that the present paper considered the interrelation between dilatation rate and density-weighted displacement speed for flames with Lewis number either equal to unity or close to unity. However, the dependence between these quantities for premixed flames with Lewis numbers significantly different from unity needs to be analysed, which will be the focus of future analyses.

## Data Availability

The data can be accessed from the corresponding author upon reasonable request.
